# Isotretinoin Use and Risk of Depression in Acne Vulgaris Patients in Riyadh, Saudi Arabia

**DOI:** 10.7759/cureus.13680

**Published:** 2021-03-03

**Authors:** Fatimah A AlGhofaili

**Affiliations:** 1 Department of Dermatology, College of Medicine, Qassim University, Buraydah, SAU

**Keywords:** depression, isotretinoin, acne vulgaris, management, beck depression inventory (bdi)

## Abstract

Background

The association between isotretinoin administration and depression in acne patients remains controversial. We aim to estimate the prevalence of depression among patients with acne vulgaris before and after treatment with isotretinoin in Riyadh, Saudi Arabia.

Methods

This was a prospective study on patients attending the King Khalid University Hospital (KKUH), a tertiary institution, who were prescribed isotretinoin for the treatment of acne vulgaris for the first time. The Beck Depression Inventory (BDI) was used to screen for depressive symptoms.

Results

A total of 179 patients were included in the study. The patients were then divided into two groups based on the treatment modality that they received: one group taking isotretinoin and the other treatment group who used other medications, including Retin-A (tretinoin) and Tazorac (tazarotene). A total of 119 patients were in the isotretinoin group with 91.6%, 2.5%, 1.7%, and 3.4% of those patients having a normal mood, mild depression, moderate depression, and severe depression scores before starting isotretinoin treatment, respectively. After three months of treatment, 94.1%, 1.7%, 0.8%, and 2.5% of patients had normal mood, mild depression, moderate depression, and severe depression, respectively. Meanwhile, after six months of treatment, 95.8%, 0.8%, 0%, and 1.7% of patients had normal mood, mild depression, moderate depression, and severe depression, respectively. The mean BDI score at the baseline was 3.31 ± 6.98 for isotretinoin and 3.17 ± 6.27 for other treatments. Compared to the baseline, patients using the isotretinoin showed a significant reduction in depression scores at three months (2.64 ± 6.17; p-value < 0.001), six months (1.99 ± 5.08; p-value < 0.001), and across all follow-up points (p-value < 0.001). Similar results were also estimated for the other treatment group, including Retin-A (tretinoin), adapalene, benzoyl peroxide, and doxycycline; however, no significant difference was noticed between the two groups (p-value = 0.885).

Conclusion

Isotretinoin treatment for acne does not appear to be associated with a statistically significant increased risk of depression in our population. Therefore, more studies are needed to understand this reflection in Saudi Arabia.

## Introduction

Acne vulgaris is recognized as an inflammatory disorder that affects the pilosebaceous units in the neck, chest, back, and face [1]. Evidence also shows that it has pleomorphic characteristics as it can be found as an inflammatory (recognized as pustules, nodules, or papules) or a non-inflammatory lesion (recognized as closed or open comedones) [2]. Acne vulgaris is a common condition in adolescence and is considered the most common skin disorder, with an estimated prevalence rate of 70% - 87% [3]. The condition has a significant impact on the affected patient and may reduce their quality of life. Such an effect can be associated with social and psychological disorders, such as depression, social phobia, low self-esteem, anxiety, and suicidal thoughts [4-5]. Besides, the economic burden is also significant as evidence shows that more than one billion dollars are spent annually on treating the condition and around 100 million dollars are being spent on acne-related products within the United States [6].

Treatment of acne vulgaris is dependent on the severity and morphology of the condition. Topical retinoids are used for mild cases, while systemic regimens that involve hormonal therapy, antibiotics, and oral retinoids are used for moderate cases. In addition, isotretinoin is the drug of choice and the most effective therapy in severe and resistant cases [1-2, 5-6]. It is a 13-cis-retinoic acid vitamin A-derivative that acts on all of the four phases of acne development [2, 7-8]. A previous meta-analysis showed that around 85% of the included patients were cured of acne after an average use of isotretinoin for four months [9]. However, many adverse events have been reported. These include skin, mouth, and nose dryness, sun sensitivity, increased levels of serum cholesterol, suicidal ideations, and depression [10].

The rate for depression after isotretinoin administration is variable and ranges between 1% and 11% [11]. However, this side effect may fade away with acne improvement. On the other hand, evidence shows that isotretinoin systemic administration is directly correlated with developing depression [12]. This was supported by previous experimental investigations which claimed that the drug plays a role in the mechanism of depression through a direct effect on the central nervous system [13]. Other researchers said that such evidence should not be counted on as a factor [12]. Although many studies have investigated the association between isotretinoin administration and depression among acne vulgaris patients, little evidence could be found regarding patients from Saudi Arabia. Consequently, we aimed to conduct this study to investigate the correlation in our Saudi population.

## Materials and methods

Sample size and data collection

This was a prospective study of 179 randomly selected new patients attending a tertiary center, King Khalid University Hospital (KKUH) who received treatment for acne vulgaris. Patients were divided into two groups based on the treatment received: the isotretinoin group and the second group using other treatments, including Retin-A (tretinoin) and Tazorac (tazarotene). Written informed consent was obtained from the study participants. An institutional review board approval was also obtained to conduct the study. We included patients who suffered from acne vulgaris and had been receiving isotretinoin or other treatment modalities for up to six months. Patients’ baseline characteristics and demographics were collected. Moreover, patients were asked if they developed any depression symptoms or not and the severity of these symptoms was assessed. Patients were divided into two groups based on the treatment modality that they received. Similar to previous studies, depression severity was measured in participants after three and six months of isotretinoin use using the Beck Depression Inventory (BDI) (1 - 10: normal; 11 - 16: mild depression; 17 - 20: borderline clinical depression; 21 - 30: moderate depression; 31 - 40: severe depression; and > 40: extreme depression) [14]. Patients were excluded if they did not fulfill the previous criteria or did not administer isotretinoin for the specified period. For patients in the isotretinoin group, oral isotretinoin was administered at a standard cumulative dose of 150 mg/kg as a daily dose of about 0.5 mg/kg.

Statistical analysis

All analyses were done using the Statistical Package for Social Sciences (SPSS), version 26 (IBM SPSS Statistics for Windows, Armonk, NY), and a p-value < 0.05 was considered significant for all tests. Continuous variables were represented as means and standard deviation, using skewness and Kurtosis tests to evaluate the normal distribution of the variables. Based on normality status, independent-samples t-test or the Mann-Whitney U test were used to compare the treatment groups. Nominal variables were presented as counts and percentages, and Chi-square or Fischer’s Exact test were used to test the differences. To test the change in BDI scores between different assessment points, the paired t-test was used to measure the change from one point to the other and the repeated measures analysis of variance (ANOVA) was used to assess the change over all three points. To assess the change of patients’ distribution, the Wilcoxon signed ranks test was used to measure the change from one point to the other, and Friedman’s test was used to assess the change over all three points.

## Results

A total of 179 patients participated in the current study of which 119 were treated with isotretinoin and 60 were managed using other treatments (Retin-A and Tazorac). The mean age for all participants was 21.35 ± 2.96 years and the majority of them were single (81.01%) and females (62.01%). About two-thirds (64.80%) of the patients had a secondary school as their highest degree obtained, while about one-third (31.28%) had university degrees and only 3.91% had diplomas. The baseline characteristics of both treatment groups were comparable, except for gender disparities (p-value < 0.001) (Table 1).

**Table 1:** Baseline Characteristics of the Included Patients *Statistically significant N: numbers; SD: standard deviation

Variables		Treatment						P-value
		Isotretinoin		Other treatments		Total		
		(N = 119)		(N = 60)		(N = 179)		
		N	%	N	%	N	%	
Age: mean (SD)		21.45 (3.01)		21.13 (2.86)		21.35 (2.96)		0.561
Gender	Female	60	50.42	51	85.00	111	62.01	< 0.001*
	Male	59	49.58	9	15.00	68	37.99	
Marital status	Married	25	21.01	9	15.00	34	18.99	0.666
	Single	94	78.99	51	85.00	145	81.01	
Education	Diploma	4	3.36	3	5.00	7	3.91	0.546
	Secondary	75	63.03	41	68.33	116	64.80	
	University	40	33.61	16	26.67	56	31.28	

The mean BDI score at the baseline was 3.31 ± 6.98 for isotretinoin and 3.17 ± 6.27 for other treatments. Compared to the baseline, patients using the isotretinoin showed a significant reduction in depression scores at three months (2.64 ± 6.17; p-value < 0.001), six months (1.99 ± 5.08; p-value < 0.001), and across all follow-up points (p-value < 0.001). Similarly, there was a significant reduction in depression scores in patients that were managed with other treatments, including three months follow-up (2.72 ± 5.76; p-value < 0.001), six months follow-up (2.47 ± 5.32; p-value < 0.001), and across all follow-up points (p-value < 0.001). However, there was no statistically significant difference when comparing the changes of the BDI score in isotretinoin and other treatment groups (p-value = 0.885) (Table 2).

**Table 2:** Changes in Depression Scores from Baseline and up to Six Months ψ Paired t-test † Repeated measures analysis of variance (ANOVA) * Statistically significant N: numbers; SD: standard deviation

Treatment	Beck Depression Inventory									
	Baseline		3 months		P-value (0 - 3 months) ψ	6 months		P-value (0-6 months) ψ	P-value (0 - 3 - 6 months)†	P-value (treatment comparison)†
	Mean	SD	Mean	SD		Mean	SD			
Isotretinoin (N = 119)	3.31	6.98	2.64	6.17	< 0.001*	1.99	5.08	< 0.001*	< 0.001*	0.885
Other treatments (N = 60)	3.17	6.27	2.72	5.76	< 0.001*	2.47	5.32	< 0.001*	< 0.001*	

Regarding depression categories, the majority of the patients were normal at baseline, whether in the isotretinoin group (91.60%) or the other treatment group (91.67%). Compared to the baseline, patients using isotretinoin showed a significant change in the distribution of depression categories at three months (p-value = 0.011), six months (p-value = 0.010), and across all follow-up points (p-value < 0.001). Nevertheless, no similar significant changes were detected among the patients managed with other treatments, when comparing the baseline to three months follow-up (p-value = 0.157), six months follow-up (p-value = 0.157), and across all follow-up points (p-value = 0.333) (Table 3).

**Table 3:** Categories of Depression Within the Study Population at Baseline and After Three and Six Months of Treatment BDI: Beck Depression Inventory; N: numbers

Depression Category		Treatment group						P-value
		Isotretinoin		Other treatments		Total		
		N	%	N	%	N	%	
BDI-baseline	Normal	109	91.60	55	91.67	164	91.62	0.978
	Mild depression	3	2.52	2	3.33	5	2.79	
	Borderline depression	1	0.84	1	1.67	2	1.12	
	Moderate depression	2	1.68	1	1.67	3	1.68	
	Severe depression	4	3.36	1	1.67	5	2.79	
	Extreme depression	0	0.00	0	0.00	0	0.00	
BDI - 3 months	Normal	112	94.12	56	93.33	168	93.85	0.893
	Mild depression	2	1.68	1	1.67	3	1.68	
	Borderline depression	1	0.84	2	3.33	3	1.68	
	Moderate depression	1	0.84	0	0.00	1	0.56	
	Severe depression	3	2.52	1	1.67	4	2.23	
	Extreme depression	0	0.00	0	0.00	0	0.00	
BDI-6 months	Normal	114	95.80	56	93.33	170	94.97	0.842
	Mild depression	1	0.84	1	1.67	2	1.12	
	Borderline depression	2	1.68	2	3.33	4	2.23	
	Moderate depression	0	0.00	0	0.00	0	0.00	
	Severe depression	2	1.68	1	1.67	3	1.68	
	Extreme depression	0	0.00	0	0.00	0	0.00	

## Discussion

Evidence shows that developing depression as a side effect following isotretinoin administration is very common. Previous studies showed that this drug is fat-soluble and can easily pass the blood-brain barrier, affecting various parts of the central nervous system involving the dopaminergic receptors, leading to depression and mood abnormalities [14-16]. Since acne vulgaris usually occurs during adolescence, management of such events is essential to provide a better quality of life (QoL) for these patients. A previous systematic review reported that isotretinoin administration in acne patients was associated with the secondary development of depression in these patients [17]. On the other hand, our results showed that isotretinoin use was associated with a significant reduction in the BDI scores over all of the follow-up points. In a systematic review conducted by Huang et al., they reported that the pooled depression scores from the included studies significantly decreased within the first four months after initiation of treatment; however, the scores began to increase within the following months [18]. Among the included studies in the same review, the authors showed that four of them indicated that the depression scores increased within the first three to four months after treatment, while another two studies reported that the scores were significantly reduced six months after treatment initiation. Our results are also consistent with Webster et al. who reported that secondary depression symptoms resolved gradually even without stopping isotretinoin administration [19]. Another recent meta-analysis also showed that isotretinoin administration was associated with decreased depressive symptoms among patients that suffered from acne [20].

Many previous studies have suggested the association between isotretinoin administration and depression, especially in patients that already suffer from depression. A previous study from Jeddah, Saudi Arabia showed that the prevalence of depression in their population was 15.8% [21]. This prevalence rate can be similar to the prevalence rate of depression among the general population [11]. We also did not find a significant difference between the isotretinoin and other treatment groups regarding the mean BDI differences. Such results suggest that the treatment of acne vulgaris, whether by isotretinoin or other treatment modalities that were used in our population, is not associated with the development of depression secondary to drug administration. Our results indicate that isotretinoin use is significantly associated with reduced BDI scores in acne treatment. This is consistent with the previous randomized controlled trial by Suarez et al. who showed that the development of depression was associated with the presence of acne, irrespective of the applied treatment modality [22]. Consequently, it can be suggested that clinicians should be aware that acne patients may suffer from depression, irrespective of the applied management modality.

Our results also indicated that no depression category was significantly prevalent over the other, either at baseline, three months, or six months of follow-up. It can also be judged that depression relieves with time after isotretinoin administration. The risk of acne relapse after isotretinoin administration might also be a contributing factor to developing depressive symptoms. A previous Saudi study showed that, among the patients that administered isotretinoin, the acne relapse rate was 45.12% in these patients [23]. Shams et al. reported that females, obese, illiterate, poor, and with severe acne suffer from reduced QoL and usually need psychological counseling together with the specified acne medication [24]. This was also supported by Dreno et al. where they reported that patients with acne relapses usually suffer from reduced QoL and loss of productivity [25]. Consequently, all the previous findings suggest that depression development is not secondary to isotretinoin administration in acne patients.

Limitations to our study are the small sample size and the design of the study as proper sampling by randomization and blinding would be better. Besides, no laboratory data were assessed, and scoring was based on patients’ self-reporting which may have impacted the results. Furthermore, the conflict of result with the literature can be attributed to response bias as patients might be thinking they will receive the treatment if if they responded in a certain way.

## Conclusions

Our study showed no correlation between developing depression and isotretinoin administration during acne treatment. On the other hand, acne management, whether by isotretinoin or by other modalities, is significantly associated with reduced depression symptoms. The results of this study is in conflict with many previous studies done on isotretinoin and depression. Therefore, more studies studying the correlation in acne patients are needed among tertiary centers in Saudi Arabia.
